# Papulopustular infantile acne treated with oral isotretinoin^⋆^

**DOI:** 10.1016/j.abd.2021.05.026

**Published:** 2023-02-15

**Authors:** Grasielle Silva Santos, Mayra Ianhez, Hélio Amante Miot

**Affiliations:** a Hospital de Doenças Tropicais Dr Anuar Auad, Goiânia, GO, Brazil; b Department of Dermatology and Radiotherapy, Universidade Estadual Paulista, Botucatu, SP, Brazil

Dear Editor,

Infantile acne is considered when it occurs between one and 16 months of age.^1^ Topical retinoids, benzoyl peroxide at low concentrations, and oral antibiotics (except tetracyclines) are used in the treatment of children.^2^

This report describes the case of a two-month-old boy who presented papules, pustules, and a cyst on the malar region, bilaterally, as well as closed and open comedones, compatible with the diagnosis of infantile acne (Fig. 1). The laboratory hormonal evaluation of the child and mother (who also had severe acne) was normal. Initially, oral erythromycin was used for two months, oral cephadroxyl for another two months, as well as the fixed combination of adapalene and benzoyl peroxide associated with non-comedogenic emollients.

**Figure 1:**
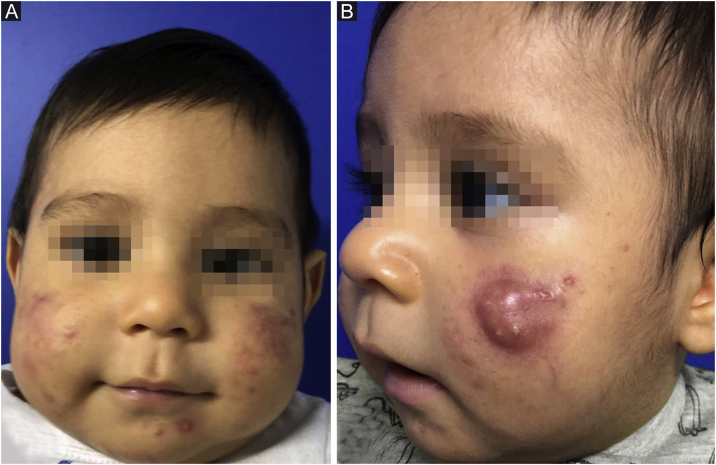
Infantile acne. (A) At two months of age, papules, pustules, and comedones on the face; (B) At seven months, even with the implemented therapy, the patient had a draining cyst, scars, and active papulopustular lesions.

Despite the prolonged use of oral antibiotics and topical medications, progression of lesions and scar formation occurred. At seven months of age, oral isotretinoin was started at a dose of 0.5 mg/kg/day (target dose 960‒1200 mg). The 10 mg capsule was frozen and half of the tablet was administered to the child in the milk.^1^

After reaching the 150 mg/kg dose nine months later and with gradual adjustment according to weight gain (up to ¾ of the tablet), there was no disease activity (Fig. 2) throughout a 12-month follow-up. During treatment, the patient had mild cheilitis and xerosis, without laboratory alterations. As post-isotretinoin maintenance therapy, the fixed combination of adapalene and benzoyl peroxide was prescribed, as well as non-comedogenic emollients.

**Figure 2:**
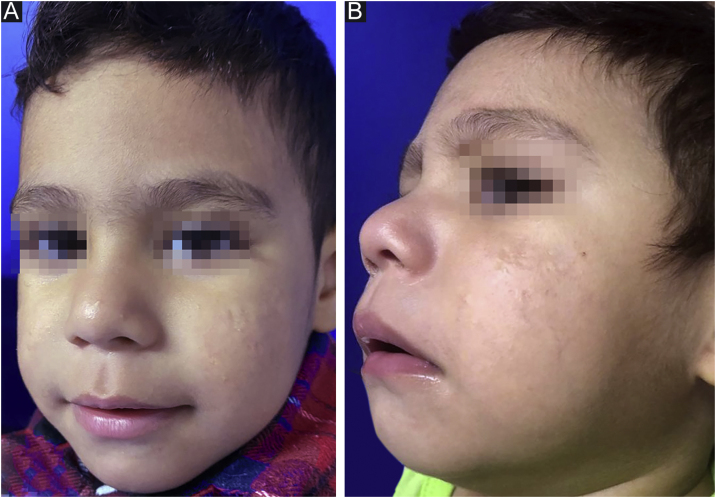
Infantile acne treated with oral isotretinoin. (A/B) One year after the end of treatment with oral isotretinoin, the patient shows residual normochromic scars.

The androgenic hormonal laboratory investigation is mandatory in cases of refractory infantile acne, although most cases are not related to underlying endocrine diseases.^1^, ^3^

Oral isotretinoin, as well as topical therapy, are off-label treatments at this age; however, the many recently published cases demonstrate not only important clinical improvement in refractory cases but also their safe use in infants.^3^, ^4^

Acitretin is used in recessive congenital ichthyosis throughout life, since birth, being the confirmation test of retinoid safety in childhood. Early closure of epiphyses in children treated with oral retinoids is a rare event, associated with previous diseases, use of high doses, or prolonged treatment.^2^ In the meantime, oral isotretinoin, when prescribed for refractory infantile acne, is a short-term treatment that requires low doses.^4^

The oral isotretinoin dose for infantile acne varies among publications between 0.2 and 2.0 mg/kg/day, with a total treatment period of five up to 14 months.^1^ According to the latest acne consensus, the cumulative dose of isotretinoin should be the one in which complete clearing of lesions is attained, with drug maintenance for two more months, in contrast to the strict recommendation of reaching 120‒150 mg/kg in all patients.^5^

Delay in the diagnosis of infantile acne is mainly due to the rarity of the disease at this age, as well as undertreatment and delay in the introduction of oral isotretinoin in these children.^1^ It is therefore important that infants with severe, chronic acne, refractory to conventional treatment, be evaluated for underlying endocrinological disorders, not delaying drug use when there is resistance to oral antibiotics as well as the formation of scars.

## Financial support

None declared.

## Authors' contributions

Grasielle Silva Santos: Design and planning of the study; data survey, analysis, and interpretation of data; drafting and editing of the manuscript; collection, analysis, and interpretation of data; intellectual participation in the propaedeutic and/or therapeutic conduct of the studied cases; critical review of the literature.

Mayra Ianhez: Design and planning of the study; data survey, analysis, and interpretation of data; critical review of important intellectual content; collection, analysis, and interpretation of data; effective participation in research orientation; intellectual participation in the propaedeutic and/or therapeutic conduct of the studied cases; critical review of the literature; approval of the final version of the manuscript.

Helio Amante Miot: Critical review of important intellectual content; analysis and interpretation of data; effective participation in research orientation; intellectual participation in the propaedeutic and/or therapeutic conduct of the studied cases; critical review of the literature; approval of the final version of the manuscript.

## Conflicts of interest

None declared.
